# Prevalence and predictors of metabolically healthy obesity in adolescents: findings from the national “Jeeluna” study in Saudi-Arabia

**DOI:** 10.1186/s12887-018-1247-z

**Published:** 2018-08-23

**Authors:** Lara Nasreddine, Hani Tamim, Aurelie Mailhac, Fadia S. AlBuhairan

**Affiliations:** 1 Department of Nutrition and Food Science, Faculty of Agricultural and Food Sciences, American University of Beirut, P.O. Box 11-0236, Riad El Solh, Beirut, Lebanon; 2 Clinical Research Institute, Biostatistics Unit, American University of Beirut Medical Center, Riad El Solh, Beirut, Lebanon; 3 Department of Pediatrics and Adolescent Medicine, AlDara Hospital and Medical Center, P.O. Box 1105, Riyadh, 11431 Saudi Arabia; 4 Department of Population, Family, and Reproductive Health, Bloomberg School of Public Health, Johns Hopkins University, Baltimore, MD USA

**Keywords:** Obesity, Adolescents, Metabolically healthy obesity, Prevalence, Predictors, Saudi Arabia, Middle-East

## Abstract

**Background:**

Obese children and adolescents may vary with respect to their health profile, an observation that has been highlighted by the characterization of metabolically healthy obesity (MHO). The objectives of this study were to examine the prevalence of MHO amongst obese adolescents in Saudi-Arabia, and investigate the anthropometric, socio-demographic, and lifestyle predictors of MHO in this age group.

**Methods:**

A national cross-sectional school-based survey (*Jeeluna*) was conducted in Saudi-Arabia in 2011–2012 (*n* = 1047 obese adolescents). Anthropometric, blood pressure and biochemical measurements were obtained. A multicomponent questionnaire covering socio-demographic, lifestyle, dietary, psychosocial and physical activity characteristics was administered. Classification of MHO was based on two different definitions. According to the first definition, subjects were categorized as MHO based on the absence of the following traditional cardiometabolic risk (CR) factors: systolic blood pressure (SBP) or diastolic blood pressure (DBP) >90th percentile for age, sex, and height; triglycerides (TG) > 1.25 mmol/L; high density lipoprotein-cholesterol (HDL-C) ≤1.02 mmol/L; glucose ≥5.6 mmol/L. The second definition of MHO was based on absence of any cardiometabolic risk factor, according to the International Diabetes Federation (IDF) criteria.

**Results:**

The prevalence of MHO ranged between 20.9% (IDF) and 23.8% (CR). Subjects with MHO were younger, less obese, had smaller waist circumference (WC) and were more likely to be females. Based on stepwise logistic regression analyses, and according to the IDF definition, body mass index (BMI) (OR = 0.89, 95% CI: 0.84–0.93) and WC (OR = 0.97, 95% CI: 0.96–0.98) were the only significant independent predictors of MHO. Based on the CR definition, the independent predictors of MHO included female gender (OR = 1.76, 95% CI: 1.29–2.41), BMI (OR = 0.97, 95% CI: 0.94–1.00), and weekly frequency of day napping (OR = 1.06, 95% CI: 1.00–1.12). Analysis by gender showed that vegetables’ intake and sleep indicators were associated with MHO in boys but not in girls.

**Conclusion:**

The study showed that one out of five obese adolescents is metabolically healthy. It also identified anthropometric factors as predictors of MHO and suggested gender-based differences in the association between diet, sleep and MHO in adolescents. Findings may be used in the development of intervention strategies aimed at improving metabolic heath in obese adolescents.

**Electronic supplementary material:**

The online version of this article (10.1186/s12887-018-1247-z) contains supplementary material, which is available to authorized users.

## Background

Pediatric obesity has become a global challenge in health care, plaguing both high and low-income nations and jeopardizing their ability to cope with the increasing cost of obesity management and treatment [1]. The Eastern Mediterranean Region (EMR), and particularly countries of the Gulf Cooperation Council (GCC), harbor one of the highest burdens of childhood obesity worldwide, with reported estimates exceeding 25% in some countries [2–4]. Childhood obesity is associated with numerous adverse health consequences, with both immediate and longer-term complications [5]. Among the immediate health risks are cardiometabolic abnormalities including insulin resistance, dyslipidemia, increased glucose levels, metabolic syndrome, and hypertension [5–7]. On the long term, childhood obesity tends to track into the adult years, increasing the risk for non-communicable diseases (NCDs), such as type 2 diabetes, cardiovascular diseases (CVDs), and certain types of cancer, while also being associated with mental health problems, such as low self-esteem and depression [8, 9].

Obesity is however being increasingly recognized as a “heterogeneous condition”, a fact that has been emphasized by the identification and characterization of metabolically healthy obesity (MHO) amongst adults [10–12]. Despite being obese, these individuals do not present any of the traditional cardiometabolic risk factors that are usually associated with obesity [10, 11]. It has been argued that this subgroup of obese subjects may have a lower mortality risk and a healthier medical prognosis, compared to their non-metabolically healthy obese counterparts [13–17]. Available studies have indicated that, amongst obese adults, the prevalence of MHO may range between 6 and 40% [10, 11, 18, 19]. Similarly, it has been suggested that obese children may also vary in terms of their health profile [12, 20–22], but MHO in the pediatric population has not been well-characterized [12]. The investigation of MHO amongst children is important for several reasons. First, given the increasing need for weight management care, it may be necessary to prioritize specialized service delivery for those individuals with the greatest cardiometabolic risk [12]. By characterizing obese individuals according to their relative health risks, those at lower cardiometabolic risk may be directed towards less intensive management services (e.g. outpatient dietitian counseling, behavioral modification etc.), while their peers at higher risk may require more intensive health services (e.g., multidisciplinary obesity treatment or drug-based management) [12]. The recognition of childhood obesity as a heterogeneous condition implies that “a menu of therapeutic options” for children (and their caregivers) would be available to address their individual health needs, an approach that is in harmony with treating obesity as a chronic disease [12, 23]. Second, given the possible protective effects of MHO on disease risk, when compared to metabolically unhealthy obese (MUO), it would be crucial to investigate and identify the characteristics that are associated with the MHO status in youth and to foster our understanding of the factors that could prevent obese subjects from developing metabolic abnormalities [24–26].

In the Kingdom of Saudi-Arabia (KSA), like in several other countries of the EMR, the rate of obesity amongst children and adolescents is following an escalating secular trend [27]. A recent national study (*Jeeluna*) conducted in KSA showed that 15.9% of adolescents were obese [28], a proportion that is considerably higher than what was reported in the early 1990s, where the prevalence of obesity was estimated at 6% in boys and 6.7% in girls aged 1–18 years [29]. This alarming trend coupled with the probable protective effect of MHO on morbidity, highlights the need to investigate and better characterize MHO in the pediatric years. This study builds on the “Jeeluna” national study to examine the proportions of obese adolescents who are metabolically healthy in KSA and to investigate socio-demographic, anthropometric, and lifestyle predictors of MHO in this age group. Due to the lack of a universal definition for MHO, two commonly used definitions will be adopted in this study to assess the prevalence and factors associated with MHO in this population [12, 30]. The selected definitions are based on traditional cardiometabolic risk factors that are easily measured and are routinely obtained in clinical practice.

## Methods

### Study design

This study is based on the national cross-sectional school-based survey (*Jeeluna*) that was conducted in KSA in 2011–2012 [28]. Details on the design, sampling protocol and data collection are published elsewhere [28]. In brief, a nationally representative sample (*n* = 12,575) of students attending intermediate/secondary schools in KSA participated in the “*Jeeluna*” study [28]. A stratified, cluster random sampling procedure was adopted with sampling occurring in all of the 13 administrative regions in the country. Within each region, several school districts exist, with a total of 42 districts in the country. Sampling occurred at the district level, ensuring that both rural, as well as urban/suburban areas were covered. Based on the student population per region, district, gender, and school level (intermediate vs. secondary), proportionate sampling was performed. For the selection of the schools, any male/female, intermediate/secondary, public/private school in a city/town in the KSA that operates during the day was eligible. Evening schools and schools that only cater for students with special needs were excluded from this study. Using a computer-based randomized sampling, schools were drawn from the list of intermediate and secondary schools enlisted with the Ministry of Education. Within the selected schools, classes were randomly chosen. Sampling was clustered whereby all students within a selected class were invited to participate in the study. An information letter describing the study objectives and protocol was sent to students and their parents.

The study protocol was approved by the institutional review board (IRB) and ethics committee at King Abdullah International Medical Research Center (KAIMRC) and the Ministry of Education (MOE). Prior to accessing the selected schools, permission was obtained from the schools’ principals. Written parental consent and student assent were obtained prior to subjects’ enrollment in the study [28]. Students were given the choice of opting out of blood sampling. Students were assured that all the responses that they provided on the questionnaire would remain anonymous and confidential.

### Study participants

For the present study, the selection of subjects from the original survey participants (*n* = 12,575) was undertaken according to the following criteria: 1) having provided blood samples; 2) not exceeding 19 years of age; and 3) being obese.

Of the total sample of 12,575 subjects, 7329 had consented to blood withdrawal and provided blood samples (response rate: 58.3%). Of those, 51 were above 19 years old and were thus excluded, yielding a sample of 7278 subjects. According to the World Health Organization (WHO) new growth standards [31], obesity was defined based on sex and age, as + 2 body mass index (BMI) *z*-scores [31]; the WHO AnthroPlus software (WHO, Geneva, Switzerland) was utilized to calculate BMI *z*-scores. To allow for comparisons with previous studies conducted in KSA, prevalence rates of obesity were also determined using the Centers for Disease Control and Prevention (CDC) 2000 criteria [32]. Accordingly, of the 7278 subjects who have provided blood samples and who were aged less than 19 years, 1179 (16.20%) were obese based on the WHO criteria and 1176 (16.16%) were obese based on the CDC criteria. For the remaining analyses, the WHO criteria were retained. In addition, subjects with missing information on blood pressure measurements, waist circumference or biochemical assessment (lipid profile; fasting glucose), were excluded (*n* = 132). Consequently, the final sample for this study included 1047 subjects.

### Data collection

Data collection included: (1) completion of self-administered multi-component questionnaire; (2) anthropometric and blood pressure measurements; and (3) blood sampling and biochemical assessment. Data collection was conducted by trained personnel who received standardized and structured training prior to the initiation of the study. Other quality control measures were implemented, including the pre-testing of the questionnaire, equipment, and data collection protocols as well as the field monitoring of data collection.

#### Completion of the multi-component questionnaire

The study questionnaire was developed based on the Youth Risk Behavior Survey [33] and the Global School-based Student Health Survey [28, 34]. Since Arabic is the native language in KSA, the Arabic version of the GSHS questionnaire was used [35]. In addition, questions adopted from the YRBS were translated to Arabic and reviewed for translational appropriateness. Cultural adaptation was introduced to the questionnaire. For instance, questions that were considered culturally inappropriate such as sexual behaviors and sexually transmitted diseases, were not included. In addition, questions related to the subject’s family, lifestyle, sleep, and health status were added. The questionnaire was reviewed for content validity by an expert panel, which included an adolescent medicine physician, a pediatrician, an epidemiologist, a public health professional, and a school health professional [36]. It is worth noting that previous investigations that were based on the same tools (Arabic version of GSHS and Arabic translation of YRBS) have reported a high internal consistency, with a Cronbach Alpha of 0.84 [37].

The questionnaire was pilot-tested on a sample of adolescents and questions or statements that were found difficult, unclear, or ambiguous to the participating adolescents were further refined or modified [36]. For instance, examples were added to clarify some questions, such as examples of main meals, snacks, vegetables, or energy drinks. In addition, the option to choose more than one response if needed was added to some questions. None of the items included in the questionnaire were found to be inappropriate or distressing to the pilot sample of adolescents and thus, none of the questions were completely eliminated [36]. The final amended version of the questionnaire was adopted for data collection (http://kaimrc.med.sa/?page_id=5036).

As such, the final version of the questionnaire covered several domains, including socio-demographic, dietary practices, sleep habits, sedentarity, physical activity, behaviors that contribute to unintentional injuries and violence, tobacco use, alcohol and substance use, history of bullying, mood, health status, and access to health services. The questionnaire was self-administered.

#### Anthropometric measurements

Anthropometric measurements were taken using standardized protocols [38] and calibrated equipment. Height was measured to the nearest 0.5 cm (cm) and body weight to the nearest 0.1 kg (kg) using an electronic scale (Omron SC100 digital scale; Omron Healthcare, Inc., Lake Forest, IL), in light indoor clothing and with bare feet or stockings [28]. BMI was calculated as the ratio of weight (kilograms) to the square of height (meters). Waist circumference was measured at the midpoint between the costal margin and iliac crest at the end of expiration using a non-elastic flexible tape measure and was recorded to the nearest millimeter (mm) [39]. Measurements were taken twice and the average was adopted.

#### Blood pressure measurements

Blood pressure (BP) was measured in the supine position by a digital BP monitor (Omron M2, Netherlands) on the right arm. After a period of rest, measurements were taken twice a few minutes apart and recorded as an average using appropriate cuff size.

#### Biochemical assessment

Licensed phlebotomists/nurses collected blood samples from the students who consented to blood withdrawal, after an 8- h fast. Blood samples were collected in serum separator tubes (BD, USA), labeled, transported at cool temperature to the hospital lab upon collection, allowed to clot for more than 15 min and then centrifuged for 10 min at 3000 rpm using a Multifuge 35R centrifuge at room temperature. The serum samples were either immediately assayed by an Architect 8000c clinical chemistry analyzer (Abbott, USA) or stored in the freezer at − 70 °C for further testing. The specimens were not stored for longer than three months due to the instability of lipids in vitro. Lipid tests were performed. In addition, glucose level was also measured on the same analyzer using the hexokinase method. Three levels of quality control were performed for each assay (Bio-Rad, USA). The patient results were stored in the Laboratory Information System (LIS) (Cerner, USA), which was interfaced with the Architect 8000c clinical chemistry analyzer. The data were then retrieved from the LIS and integrated within the *Jeeluna* database.

### Definition of metabolically healthy obesity (MHO)

We applied two different definitions to examine the prevalence and factors associated with MHO. The first definition was the one proposed by Prince et al. [12], according to which subjects were categorized as MUO or MHO, based on the presence or absence of the following four traditional cardiometabolic risk (CR) factors (MHO: 0 risk factor; MUO: > 1 risk factors): systolic blood pressure (SBP) or diastolic blood pressure (DBP) >90th percentile for age, sex, and height [40]; triglycerides (TG) > 1.25 mmol/L [41, 42]; high density lipoprotein-cholesterol (HDL-C) ≤ 1.02 mmol/L; and glucose ≥5.6 mmol/L [12].

The second definition of MHO was based on the International Diabetes Federation (IDF) criteria. Accordingly, participants were dichotomized as MHO or MUO based on the presence or absence of the following risk factors: amongst those aged between 10 and 16 years old: SBP ≥130 or DBP ≥85 mmHg; TG ≥1.7 mmol/L; HDL-C < 1.03 mmol/L; glucose ≥5.6 mmol/L and waist circumference (WC) ≥90th percentile for age and sex or adult cut-off if lower [30, 43]. Amongst those aged between 16 and 19 years old: SBP BP ≥130 or diastolic BP ≥85 mmHg; TG ≥ 1.7 mmol/L; HDL-C < 1.03 mmol/L in males and < 1.29 mmol/L in females; glucose ≥5.6 mmol/L and WC ≥ 94 cm for males and ≥ 80 cm for girls.

### Data analysis

Identical analyses were conducted for both the IDF and CR definitions. Subjects’ characteristics were described with number and percent for categorical variables and mean and standard deviation for continuous ones. The chi-square test and the independent t-test were used to assess statistically significant differences between groups (MHO vs MUO). We set statistical significance at a two-sided *p*-value of < 0.05. The associations between MHO status and subjects’ characteristics (socio-demographic, anthropometric, dietary, physical activity, sleep, smoking and psychosocial characteristics) were further assessed by creating a logistic regression model with MHO as a dependent variable, and with adjustment for age and sex. Stepwise logistic regression analyses (forward selection) was conducted to determine the independent predictors of MHO. The potential predictors that were entered into the final multivariate regression models included those that were significantly associated with MHO, based on either the IDF or CR definitions, in the age and sex adjusted regression analyses. Moreover, given that available evidence suggests a sleep-gender interaction [25, 44], we tested for a potential interaction between gender and sleep related variables in our study sample. An interaction term was created for gender and number of hours of sleep during weekdays, for gender and number of hours of sleep during week-ends, and for gender and frequency of daytime napping. The interaction terms were entered separately into the logistic regression models, and the interaction was assessed for both models (Model-IDF definition and Model-CR definition). Whenever significant interaction was found, results were reported for both genders separately. Results were reported as odds ratio (OR), and 95% confidence interval (95% CI). We performed all data management and analyses using the Statistical Analysis Software (Version 9.1; 2004).

## Results

Of the study sample, 62.6% were boys and 37.4% were girls. The age of the study participants ranged between 10 and 19 years, with a mean of 15.9 years (±1.9) in boys and 15.6 years (±1.8) in girls. The large majority of the students participating in the study were of Saudi nationality (84.9%) (data not shown).

As shown in Tables 1, 219 subjects out of 1047 (20.9%, 95% confidence interval (CI): 18.4–23.4) were categorized as MHO based on the IDF definition and 249 out of 1047 (23.8%, 95% CI: 21.2–26.4) were categorized as MHO based on the CR definition. The results showed that 12.8% of the participants were classified as being MHO based on both definitions (IDF and CR), while 68.1% were categorized as MUO by both categorizations (data not shown).

**Table 1:** Socio-demographic, anthropometric and cardiometabolic status amongst adolescents (*n* = 1047) in KSA by MUO or MHO status

	IDF			CR		
	MUO (*n* = 828)	MHO (*n* = 219)	p-value	MUO (*n* = 798)	MHO (*n* = 249)	p-value
Socio-demographic						
Age (years)	15.88 ± 1.85	15.41 ± 1.86	0.0008	15.77 ± 1.85	15.81 ± 1.91	0.81
Gender						
Males	534 (64.5)	121 (55.3)	0.01	520 (65.2)	135 (54.2)	0.002
Females	294 (35.5)	98 (44.8)		278 (34.8)	114 (45.8)	
Father’s level of education						
Elementary or less	145 (19.7)	35 (18)	0.84	128 (18.1)	52 (23.2)	0.17
Intermediate-high school	331 (45)	91 (46.7)		330 (46.7)	92 (41.1)	
University or higher	259 (35.2)	69 (35.4)		248 (35.1)	80 (35.7)	
Mother’s level of education						
Elementary or less	251 (33.7)	57 (28.9)	0.45	235 (32.8)	73 (32.2)	0.82
Intermediate- high school	291 (39)	81 (41.1)		285 (39.8)	87 (38.3)	
University or higher	204 (27.4)	59 (30)		196 (27.4)	67 (29.5)	
Anthropometric						
Height (cm)	162.78 ± 10.43	158.08 ± 11.42	< 0.0001	162.2 ± 10.97	160.5 ± 10.2	0.03
Weight (kg)	88.45 ± 17.4	77.11 ± 14.27	< 0.0001	86.94 ± 17.63	83.31 ± 16.4	0.004
BMI (kg/m^2^)	33.28 ± 5.43	30.76 ± 4.25	< 0.0001	32.93 ± 5.44	32.19 ± 4.79	0.04
BMI Z score	2.82 ± 0.75	2.5 ± 0.57	< 0.0001	2.78 ± 0.74	2.67 ± 0.66	0.02
WC (cm)	93.32 ± 18.78	80.97 ± 16.84	< 0.0001	91.08 ± 19.55	89.65 ± 17.37	0.27
Elevated WC	447 (54.0)	0 (0.0)	< 0.0001	357 (44.7)	90 (36.1)	0.02
Cardiometabolic						
SBP (mm Hg)	127.66 ± 11.96	118.05 ± 7.8	< 0.0001	128.6 ± 11.32	116.19 ± 8.08	< 0.0001
DBP (mm Hg)	72.84 ± 10.85	67.75 ± 8.7	< 0.0001	73.37 ± 10.88	66.64 ± 7.9	< 0.0001
TC (mmol/L)	4.35 ± 0.78	4.24 ± 0.62	0.04	4.36 ± 0.77	4.2 ± 0.66	0.001
HDL-C (mmol/L)	1.13 ± 0.23	1.29 ± 0.2	< 0.0001	1.13 ± 0.24	1.27 ± 0.19	< 0.0001
LDL-C (mmol/L)	2.79 ± 0.7	2.63 ± 0.6	0.001	2.8 ± 0.69	2.63 ± 0.64	0.0007
TG (mmol/L)	1.26 ± 0.76	0.87 ± 0.29	< 0.0001	1.3 ± 0.76	0.79 ± 0.21	< 0.0001
Glucose (mmol/L)	4.62 ± 0.96	4.41 ± 0.67	0.0003	4.63 ± 0.97	4.4 ± 0.67	< 0.0001

Numbers are presented as Mean ± SD or as proportions, n(%)

Abbreviations: BMI: Body mass index; cm: Centimeters; CR: Cardiovascular risks; DBP: Diastolic blood pressure; HDL-C: High density lipoprotein-cholesterol; IDF: International Diabetes Federation; kg: Kilograms; kg/m^2^: Kilograms per squared meters; KSA: Kingdom of Saudi Arabia; LDL-C: Low density lipoprotein-cholesterol; MHO: Metabolically healthy obesity; mm Hg: Millimeters of mercury; mmol/L:Millimoles per liter; MUO: Metabolically unhealthy obesity; SBP: Systolic blood pressure; SD: Standard deviation; TC: Total cholesterol; TG: Triglycerides; WC: Waist circumference

Gender disparities were noted in the proportions of MHO and MUO, according to both definitions (Table 1). Significant differences in age were observed with the IDF definition only, whereby subjects with MHO were younger. Across both the IDF and CR categories, the MHO group was significantly shorter, lighter, and less obese (lower BMI values and lower BMI z scores) than their MUO peers (Table 1). WC (cm) was significantly lower amongst MHO subjects based on the IDF definition. Similarly, the proportion of subjects with elevated WC was significantly lower amongst MHO subjects, based on the CR categorization. As expected, cardiometabolic risk factors were in the less healthy direction in the MUO group.

Table 2 shows the dietary and lifestyle characteristics of the study population. In approximately 60% of the study subjects, the daily frequency of fruits’ consumption was nil, while another equal proportion reported no consumption of milk. Similarly, 60% of the study subjects reported an intake of two or more soft drinks per day. Around half of the adolescents reported irregular breakfast consumption, no intake of vegetables, no exercise at school, and inadequate sleep on week days as well as week-ends. More than 80% of the study population reported screen time exceeding 2 h per day. There were no significant differences between the MHO and MUO groups in dietary and lifestyle characteristics, except for the weekly frequency of day napping, which was found to be significantly higher in the MHO group based on the CR definition. Psychosocial variables were also investigated amongst the study subjects (Additional file 1: Table S1). There were no significant differences in any of the psychosocial variables between MHO and MUO groups, according to both definitions.

**Table 2:** Dietary and lifestyle characteristics amongst adolescents (n = 1047) in KSA by MUO or MHO status

	IDF			CR		
	MUO (*n* = 828)	MHO (*n* = 219)	*p*-value	MUO (*n* = 798)	MHO (*n* = 249)	*p*-value
Dietary habits						
Regular breakfast consumption during the past month						
*No*	360 (44)	104 (48.2)	0.27	353 (44.7)	111 (45.1)	0.92
*Yes*	459 (56)	112 (51.9)		436 (55.3)	135 (54.9)	
Frequency of snacks consumption/d						
0	444 (54.1)	124 (57.4)	0.39	437 (55.3)	131 (53.3)	0.5
1	220 (26.8)	48 (22.2)		207 (26.2)	61 (24.8)	
≥ 2	157 (19.1)	44 (20.4)		147 (18.6)	54 (22)	
Frequency of fruits consumption/d						
0	503 (61.1)	134 (61.5)	0.51	475 (59.9)	162 (65.3)	0.31
1	125 (15.2)	27 (12.4)		119 (15)	33 (13.3)	
≥ 2	195 (23.7)	57 (26.2)		199 (25.1)	53 (21.4)	
Frequency of vegetables consumption/d						
0	385 (46.8)	100 (46.1)	0.59	357 (45)	128 (51.8)	0.14
1	266 (32.3)	65 (30)		263 (33.2)	68 (27.5)	
≥ 2	172 (20.9)	52 (24)		173 (21.8)	51 (20.7)	
Frequency of soft drinks consumption/d						
≤ 1	320 (38.8)	95 (44.2)	0.15	317 (39.9)	98 (40.0)	0.98
≥ 2	504 (61.2)	120 (55.8)		477 (60.1)	147 (60.0)	
Frequency of energy drinks consumption/d						
0	657 (79.8)	170 (78.3)	0.63	629 (79.2)	198 (80.5)	0.67
≥ 1	166 (20.2)	47 (21.7)		165 (20.8)	48 (19.5)	
Frequency of milk consumption/d						
0	468 (57.3)	129 (60.3)	0.73	449 (57.1)	148 (60.4)	0.66
1	223 (27.3)	54 (25.2)		215 (27.4)	62 (25.3)	
≥ 2	126 (15.4)	31 (14.5)		122 (15.5)	35 (14.3)	
Frequency of fast food consumption/week	2.03 ± 1.94	1.87 ± 1.78	0.29	2.03 ± 1.95	1.9 ± 1.76	0.36
Physical Activity and sedentarity						
Exercise in school						
No	486 (59.7)	130 (60.8)	0.78	464 (59)	152 (62.8)	0.29
Yes	328 (40.3)	84 (39.3)		322 (41)	90 (37.2)	
Frequency of exercise for at least 30 mn during the past week	1.76 ± 2.34	1.64 ± 2.3	0.51	1.8 ± 2.36	1.52 ± 2.2	0.11
Screen time						
≤ 2 h/d	132 (18.7)	37 (19.5)	0.81	126 (18.5)	43 (20)	0.63
> 2 h/d	573 (81.3)	153 (80.5)		554 (81.5)	172 (80)	
Sleep						
Number of hours of sleep per night, during week days						
Adequate	325 (40.3)	100 (46.3)	0.11	312 (40.1)	113 (46.5)	0.07
Inadequate	481 (59.7)	116 (53.7)		467 (60)	130 (53.5)	
Number of hours of sleep per night, during weekends						
Adequate	334 (48.1)	92 (50.8)	0.52	323 (48.4)	103 (49.5)	0.78
Inadequate	360 (51.9)	89 (49.2)		344 (51.6)	105 (50.5)	
Number of days went to sleep during the day in the past week	3.79 ± 2.86	3.86 ± 2.95	0.73	3.69 ± 2.89	4.14 ± 2.83	0.03
Smoking						
Smoking cigarettes during the past month						
No	746 (91.2)	196 (91.2)	0.99	715 (91)	227 (91.9)	0.65
Yes	72 (8.8)	19 (8.8)		71 (9)	20 (8.1)	
Smoking Shisha during the past month						
No	755 (92.4)	200 (93.5)	0.6	726 (92.6)	229 (92.7)	0.95
Yes	62 (7.6)	14 (6.5)		58 (7.4)	18 (7.3)	

Screen time was assessed based on the time spent watching television, using the internet, chatting and playing videogames

Adequate sleep defined as a minimum of 9 h for 10–12 year old adolescents and as a minimum of 8 h for those aged13 years or older [82]

Abbreviations: CR: Cardiovascular risks; d: Day; IDF: International Diabetes Federation; KSA: Kingdom of Saudi Arabia; MHO: Metabolically healthy obesity; mn: Minutes; MUO: Metabolically unhealthy obesity

The predictors of MHO status, after adjustment for age and sex, are shown in Table 3. Across both definitions, female gender was associated with higher odds of MHO (OR = 1.43, 95% CI: 1.06–1.94 based on IDF; OR = 1.59, 95% CI: 1.19–2.12 based on CR). Age was significantly inversely associated with MHO, based on the IDF categorization (OR = 0.88; 95% CI: 0.81–0.95). Compared to the lowest level of fathers’ education (elementary or less), an intermediate or high-school educational level was associated with lower odds of MHO based on the CR definition, and the association was close to significance (OR = 0.68, 95% CI: 0.46–1.02). Across both definitions, there was a significant inverse association between MHO, weight, BMI and BMI-z score, with the latter being the strongest anthropometric predictor of MHO. There was also a negative association between WC (cm) and MHO based on the IDF definition, while elevated WC was associated with lower odds of MHO based on the CR categorization. The daily frequencies of vegetable and soft drink consumption were associated with lower odds of MHO, based on the CR and IDF definitions, respectively. Meeting the sleep recommendations during weekdays as well as the weekly frequency of day napping, were positively associated with MHO. Across both definitions, there was no association between MHO and any of the psychosocial variables under investigation (data not shown).

**Table 3:** Association of socio-demographic, anthropometric, dietary and lifestyle characteristics with MHO after age and sex adjustment

	IDF-MHO		CR-MHO	
	OR (95% CI)*	*p*-value	OR (95% CI)*	*p*-value
Socio-demographic				
Age (years)	0.88 (0.81–0.95)	0.001	1.02 (0.94–1.10)	0.65
Gender				
Males	reference		reference	
Females	1.43 (1.06–1.94)	0.02	1.59 (1.19–2.12)	0.002
Father’s level of education				
Elementary or less	reference		reference	
Intermediate or high school	1.13 (0.73–1.76)	0.58	0.68 (0.46–1.02)	0.06
University or higher	1.07 (0.68–1.70)	0.76	0.83 (0.55–1.25)	0.37
Mother’s level of education				
Elementary or less	reference		reference	
Intermediate or high school	1.18 (0.80–1.73)	0.4	0.99 (0.69–1.42)	0.95
University or higher	1.23 (0.82–1.87)	0.32	1.14 (0.77–1.68)	0.5
Anthropometric				
Weight (Kg)	0.95 (0.94–0.96)	< 0.0001	0.99 (0.98–1.00)	0.01
BMI (Kg/m^2^)	0.87 (0.83–0.91)	< 0.0001	0.96 (0.93–1.00)	0.03
BMI Z score	0.36 (0.26–0.51)	< 0.0001	0.78 (0.62–0.98)	0.03
WC (cm)	0.97 (0.96–0.98)	< 0.0001	1.00 (0.99–1.01)	0.74
Elevated WC	NA		0.74 (0.55–1.00)	0.05
Dietary Habits				
Regular breakfast consumption in the past month				
*No*	reference		reference	
*Yes*	0.90 (0.66–1.23)	0.51	1.09 (0.81–1.47)	0.55
Frequency of snacks consumption/d				
≥ 3	reference		reference	
2	0.77 (0.49–1.22)	0.27	0.79 (0.52–1.21)	0.28
≤ 1	1.01 (0.68–1.50)	0.96	0.81 (0.56–1.17)	0.27
Frequency of fruits consumption/d				
0	reference		reference	
1	0.78 (0.49–1.25)	0.3	0.83 (0.54–1.27)	0.38
≥ 2	1.04 (0.73–1.49)	0.81	0.81 (0.57–1.15)	0.24
Frequency of vegetables consumption/d				
0	reference		reference	
1	0.89 (0.63–1.27)	0.54	0.70 (0.50–0.98)	0.04
≥ 2	1.12 (0.76–1.65)	0.56	0.83 (0.57–1.20)	0.32
Frequency of soft drinks consumption/d				
≥ 2	reference		reference	
≤ 1	0.73 (0.54–1.00)	0.05	0.94 (0.70–1.26)	0.66
Frequency of power drinks consumption/d				
≥ 1	reference		reference	
0	0.86 (0.60–1.25)	0.43	1.04 (0.72–1.49)	0.84
Frequency of milk drinks consumption/d				
0	reference		reference	
1	0.85 (0.59–1.21)	0.36	0.88 (0.63–1.24)	0.48
≥ 2	0.87 (0.56–1.35)	0.53	0.91 (0.60–1.39)	0.67
Frequency of fast food consumption/week	0.97 (0.90–1.06)	0.50	0.98 (0.91–1.06)	0.58
Physical Activity and Sedentarity				
Exercise in school				
No	reference		reference	
Yes	1.19 (0.81–1.74)	0.37	1.13 (0.79–1.61)	0.51
Screen Time				
> 2 h/day	reference		reference	
≤ 2 h/day	1.01 (0.67–1.52)	0.96	1.08 (0.73–1.59)	0.71
Frequency of exercise for at least 30 mn in the past week	0.98 (0.91–1.04)	0.45	0.96 (0.90–1.02)	0.21
Sleep				
Number of sleep hrs per night, during week days				
Inadequate	reference		reference	
Adequate	1.31 (0.97–1.78)	0.08	1.34 (1.00–1.80)	0.05
Number of sleep hrs per night, during weekends				
Inadequate	reference		reference	
Adequate	1.10 (0.79–1.53)	0.57	1.03 (0.75–1.41)	0.86
Number of days went to sleep during the day in the past week	1.02 (0.97–1.08)	0.44	1.05 (1.00–1.11)	0.05
Smoking				
Smoking cigarette during the past month				
Yes	reference		reference	
No	0.75 (0.43–1.30)	0.3	1.00 (0.59–1.71)	0.99
Smoking shisha during the past month				
Yes	reference		reference	
No	0.88 (0.47–1.63)	0.68	0.88 (0.50–1.55)	0.66

*Analyses were adjusted for age and sex except for BMI Z score since this variable already adjusts for inter-individual differences in age and sex

**Abbreviations:** BMI: Body mass index; CI: Confidence interval; cm: Centimeters; CR: Cardiovascular risks; d: Day; hrs: Hours; IDF: International Diabetes Federation; kg: Kilograms; kg/m^2^: Kilograms per squared meters; MHO: Metabolically healthy obesity; mn: Minutes; OR: Odds ratio; WC: Waist circumference

Stepwise logistic regression was carried out to determine the independent predictors of MHO (Table 4). The model included the variables that were significantly associated with MHO (for either definition) after age and sex adjustment. As such, the final model included age, gender, BMI (kg/m^2^), WC (cm), father’s level of education, frequency of vegetable consumption per day, frequency of soft drinks’ consumption per day, sleep hours per night, and daytime napping. It is important to note that, since the final model included both age and sex, we selected BMI (kg/m^2^) instead of BMI-z score, given that the latter already adjusts for inter-individual differences in age and sex. In addition, since significant interactions were found between gender, the number of sleep hours during week-days, and the frequency of napping, analyses were performed for boys and girls separately as well as for the total study population. Based on the IDF definition, BMI and WC were the only significant independent predictors of MHO in the overall sample. Based on the CR categorization, the significant independent predictors of MHO included female gender, BMI and the weekly frequency of day napping. Gender-disparities in MHO predictors were noted. MHO defined as per the IDF criteria was associated with BMI and WC in both genders, but in boys, the predictors also included the weekly frequency of consuming 2 vegetables per day (in comparison with a reference intake of 0/day). The weekly frequency of day napping as well as meeting the sleep recommendations during week-days also reached borderline significance in boys, but not in girls. Based on the CR categorization, the significant independent predictors of MHO included BMI and the frequency of soft drink consumption in girls, and father’s level of education in boys.

**Table 4:** Independent associations of socio-demographic, anthropometric, and lifestyle characteristics with MHO status

Model-IDF definition*	OR (95%CI)	*p*-value
Among All		
BMI (kg/m^2^)	0.89 (0.84–0.93)	< 0.0001
WC (cm)	0.97 (0.96–0.98)	< 0.0001
Model-CR definition*		
Female Gender	1.76 (1.29–2.41)	0.0004
BMI (kg/m^2^)	0.97 (0.94–1.00)	0.06
Number of days went to sleep during the day in the past week	1.06 (1.00–1.12)	0.04
Among Boys		
Model-IDF definition*		
BMI (kg/m^2^)	0.91 (0.85–0.96)	0.001
WC (cm)	0.97 (0.96–0.99)	< 0.0001
Frequency of vegetable consumption/d, ≥2/day	1.77 (1.07–2.91)	0.02
Number of sleep hours during week days, adequate	1.51 (0.99–2.35)	0.07
Number of days went to sleep during the day in the past week	1.07 (0.99–1.16)	0.08
Model-CR definition*		
Father’s level of education, intermediate-high school	0.60 (0.39–0.92)	0.02
Among Girls		
Model-IDF definition*		
BMI (kg/m^2^)	0.84 (0.77–0.92)	0.0001
WC (cm)	0.97 (0.96–0.99)	0.0005
Model-CR definition*		
BMI (kg/m^2^)	0.95 (0.89–1.00)	0.06
Frequency of soft drinks consumption/d, ≤1/day	0.49 (0.30–0.81)	0.006

*For each definition of MHO, the variables included in the model were: age (by unit increase of 1 year), gender (reference: male), BMI (by unit increase of 1 kg/m^2^), waist circumference (by unit increase of 1 cm), father’s level of education (reference: lowest), frequency of vegetables’ consumption per day (reference: 0), Frequency of soft drinks’ consumption per day (reference: ≥2), Number of sleep hours per night, during week days (reference: < 9 h for 6–12 years old; < 8 h for those aged 13 years and above), number of days went to sleep during the day (by unit increase of 1 day). Since the model included age and gender as variables, we included BMI in the regression model instead of BMI-zcore, given that the latter already adjusts for inter-individual differences in age and sex

**Abbreviations:** BMI: Body mass index; CI: Confidence interval; cm: Centimeters; CR: Cardiovascular risks; hrs: Hours; IDF: International Diabetes Federation; kg/m^2^: Kilograms per squared meters; MHO: Metabolically healthy obesity; OR: Odds ratio; WC: Waist circumference

## Discussion

This study is the first to investigate MHO amongst adolescents in the Eastern Mediterranean Region. The study showed that approximately one in five obese adolescents in KSA was identified as metabolically healthy, despite being obese. In agreement with previous reports [12, 21, 45–53], subjects with MHO were significantly younger, less obese, had smaller waist circumference and were more likely to be females. In addition, sleep habits and vegetable intake were found to be significantly associated with MHO in the study population, particularly in boys. Interestingly, the factors that predicted MHO varied depending on the definition that was used to identify subjects as MHO or MUO.

The study findings showed that the prevalence of MHO amongst obese adolescents in KSA (20.9–23.8%) falls within the range reported in the literature (3.9–49.3%) [12, 22, 45–56]. Caution must however be exerted when comparing prevalence estimates of MHO given that various studies may have adopted different definitions and that some studies included both overweight and obese subjects when assessing MHO. In certain studies, the definition of MHO was based on the presence of insulin resistance as estimated by Homeostasis Model Assessment (HOMA) [12, 21, 22, 48, 50], or as the presence of less than 2 cardiometabolic risk factors [21, 45–47, 54], while in others, including the present study, MHO was identified based on the absence of any cardiometabolic risk factor [12, 22, 48, 49, 51–53, 55, 56]. In addition, the criteria adopted to define individual cardiometabolic abnormalities were often discrepant between studies, and included those proposed by the IDF, the National Cholesterol Education Program (NCEP), the modified Adult treatment Panel III (ATPIII), as well as other ethnic specific criteria. Based on the CR definition proposed by Prince et al. (2014) [12], the prevalence of MHO obtained in this study (23.8%) was lower than the one reported amongst 8–17 year old overweight and obese Canadian children (MHO: 31.5%) and amongst obese German children (mean age: 11.6 ± 2.8 years) (MHO: 49.3%) [51]. The younger age of the children participating in these studies and the fact that both overweight and obese children were included in the study by Prince et al. (2014) [12] may explain the higher proportion of MHO in these studies, compared to our results. Based on the IDF definition, the prevalence of MHO obtained in this study (20.9%) was similar to the one reported amongst obese children and adolescents (10–18 years old) in Belgium (18.6%) [22], and lower than the estimate reported amongst obese youth aged 8–18 years in Austria (30.7%) [55]. Importantly, the results of this study highlighted poor agreement between definitions in classifying subjects as MHO, whereby only 12.8% of the participants were classified as MHO based on both the IDF and CR definitions. Poor agreement between various MHO definitions has also been described by other studies [12, 48], underscoring the need for a harmonized definition for the identification of MHO in clinical as well as research settings.

It remains important to note that, at the time of this study, the MHO subjects were healthier than their MUO peers based on measures of traditional cardiometabolic health risk, but it is unknown whether this discrepancy would remain stable over time or whether it may be extrapolated to other health domains (such as musculoskeletal, respiratory) or whether the inclusion of other indicators of cardiovascular health (e.g., apo B, inflammatory markers, insulin resistance) would impact the MHO prevalence estimates obtained in this study [12]. It has been debated that MHO may not be a stable phenotype and there are unanswered questions on whether it represents a transient phenotype, changing with age, from childhood into adulthood [57]. However, based on the Bogalusa Heart Study, where 1098 individuals had participated both as children (5–17 years) and as adults (24–43 years), Li et al. (2012) showed that MHO children had favorable cardiometabolic profiles and carotid intima media thickness (CIMT) in adulthood compared with MUO children, thus providing evidence that the MHO phenotype starts in childhood and continues into adulthood [58] .

In this study, and in concordance with other reports, there were differences in how MHO related to adiposity, socio-demographic and lifestyle predictors, depending on the classification used to define MHO. First, WC was significantly associated with MHO, based on both the IDF and CR definitions, independently of age and sex, thus highlighting the importance of measuring WC in clinical settings. However, in the fully adjusted model, WC remained an independent predictor of MHO based on the IDF definition only, and not the CR definition. Similar results were obtained by other studies that have adopted the CR definition. For instance, Prince et al. (2014) has shown that WC was no longer significantly related with MHO in Canadian children, after adjustment for lifestyle factors [12]. In addition, the results of this study showed that, based on both definitions, BMI- z score was the strongest predictor of MHO amongst adolescents in KSA, and that, after adjustment for dietary and lifestyle factors, BMI was more strongly associated with MHO, compared to WC. These results are in agreement with those reported by a longitudinal study amongst adolescents, where BMI and its changes over time were more strongly related to cardiovascular factors compared with WC [51]. It is important to acknowledge that WC may not always be reflective of visceral fat as it is not able to differentiate between subcutaneous fat in the abdominal area and visceral fat accumulation [59]. Taken together, these findings suggest that the screening of an obese adolescent may include WC as a proxy of abdominal obesity [60, 61], in conjunction with BMI which may be able to better predict metabolic health in this age group. It has in fact been argued that BMI is one of the most consistent determinants of MHO in adolescents [12, 52, 56] and that MHO status may not be really found at higher levels of obesity [21].

In this study, we found an association between the frequency of consumption of vegetables and MHO amongst boys, but not girls. Gender-based differences in the association of diet composition with MHO have been previously reported amongst adults [44] but no studies have examined these differences in children and adolescents. Such gender-based disparities may reflect differences in physiology or in the reporting of dietary intakes between sexes. The combination of phytochemicals, antioxidants, and dietary fiber brought by a diet rich in vegetables may decrease oxidative stress, mitigate the inflammatory response, improve insulin sensitivity and decrease cardio-metabolic risk, which may explain our study findings [62]. It is worth noting that some studies have reported a positive association of MHO with the intake of milk and fruits, and an inverse association with the consumption of soft drinks [47, 50, 54], while other studies found no association between MHO and food groups’ intakes [12, 48]. Surprisingly, our results showed that, in girls, a lower intake of soft drinks was associated with lower odds of MHO. This may be due to the fact that adolescent girls, and particularly those with high adiposity, tend to under-report their intakes of energy-dense, nutrient-poor foods [63–65]. It is important to note that the questionnaire adopted in this study was qualitative in nature, and did not obtain quantitative information on portions or serving sizes usually consumed. In addition, the questionnaire did not allow for the estimation of energy and macronutrient intakes, and hence differences between MHO and MUO groups in this respect, could not be investigated.

Based on the CR definition, the results showed that meeting the recommended number of sleep hours per night was associated with higher odds of MHO in the total sample, after adjustment for age and sex. This sleep indicator was also associated with MHO in boys, based on the IDF definition. These findings are in line with those reported by Li et al. (2015) amongst Chinese children and adolescents, where MHO subjects had significantly longer sleep hours, and with those reported by Spruyt et al. (2010), where shorter sleep durations among children in the United States (US) were strongly associated with adverse metabolic outcomes such as higher plasma levels of insulin, low density lipoprotein (LDL) and high sensitivity C-reactive protein [50, 66]. Interestingly, the results of our study have also shown that the weekly frequency of day napping was an independent positive predictor of MHO status, particularly in boys. Studies on the association between day napping and metabolic health are scarce. In adults, longer napping durations (> 60–90 mn) were associated with higher risk of Metabolic syndrome and incidence of coronary heart disease, while this association was not observed for shorter nap durations (< 30–60 mn) [67–69]. In high school students, afternoon or evening naps, as assessed by actigraphy, were associated with higher levels of interleukin 6, while this association was not found for morning naps [70]. The same study has reported that diary-reported napping was not associated with any inflammatory marker. In addition, although some studies have suggested that daytime naps may be associated with reduced nocturnal sleep and with increased food craving amongst adolescents [71], others have found no association between daytime sleep and increased risk of adiposity in children and adolescents [72, 73]. It has been proposed that nighttime sleep and naps serve different physiological functions. Naps may in fact reduce daytime psychosocial stress and cortisol levels, which may, at least partly explain the observed associations in our study [72, 74, 75].

It is of interest that, in our study, sleep indicators were associated with MHO in boys only, and not in girls. Gender-based differences in the association between sleep and metabolic health have been previously described amongst adults [25], but few studies have tackled this association in adolescents. In a nationally representative survey of 7–15 year old children and adolescents, short sleep duration was associated with elevated waist circumference, and this association was observed amongst boys only [76]. In a study conducted on children and adolescents aged 6–20 years, short sleep duration was associated with lower resting energy expenditure in boys and with higher leptin levels in girls [77, 78]. These results suggest a possible gender difference in the impact of sleep duration on hormonal and physiologic parameters during childhood and adolescence [78]. Alternatively, the gender-based disparities in the association between sleep and MHO may reflect differences in lifestyle-related factors. In fact, the widespread use of videogames and technology among teenage boys, may delay the onset of sleep, possibly introducing daytime napping as well [79]. Consequently, this group is at higher risk of disruption of the normal circadian rhythmicity related to sleep and the hormonal systems involved in metabolic regulation [79]. Taken together, our findings highlight the need for the integration of sleep in the development of effective prevention, treatment, and intervention programs targeting adolescent obesity and related metabolic abnormalities [76, 80]. The inclusion of sleep questions in health assessments can provide a clear picture of whether the adolescent has good or poor sleeping habits and help in planning for lifestyle and behavior modification interventions when needed [80].

In the present study, there was no association between physical activity, sedentary behavior, and MHO amongst adolescents. The link between physical activity and MHO status in youth is not well understood, since only few studies have examined this association. Prince et al. (2014) [12] showed that higher physical activity was independently associated with MHO amongst Canadian children, while Camhi et al. (2013), Heinzle et al. (2015) and Senechayl et al. (2013) reported no associations between physical activity, screen time and MHO amongst US and Canadian adolescents [21, 46, 52]. The lack of association between MHO and physical activity in the present study may be due to the low prevalence of physical activity amongst obese adolescents in KSA whereby the frequency of engaging in physical activity for at least 30 min was less than 2 times per week in this population group. Alternatively, other factors such as cardiorespiratory or musculoskeletal fitness, which may offer additional insight as to why some obese adolescents experience metabolic abnormalities while others do not [45, 52], were not assessed in this study.

The strengths of this study included the large sample and the national representativeness of the study population. Anthropometric measurements were obtained using standardized protocols rather than being self-reported. The findings of this study should however be interpreted in light of the following limitations. First, the study instrument was self-administered which may be associated with recall bias, and a high cognitive burden [81]. However, the questionnaire underwent several rounds of expert review and was pilot-tested for clarity, appropriate wording and comprehension amongst the target respondent group, i.e. adolescents in KSA. Second, pubertal stage and the levels of sex hormones, which may affect the cardiometabolic profile, were not assessed in this study [22, 48, 50, 51]. In addition, direct measures of adiposity, such as fat mass, percent body fat and visceral fat, which may play a crucial role in the pathogenesis of metabolic abnormalities, were not obtained. It is also important to note that physical activity and dietary assessment were not investigated using objective measurements, but were self-reported based on questions that were formulated in congruence with those included in the Youth Risk Behavior Survey [33] and the Global School-based Student Health Survey [28, 34], with cultural adaptation. It is worth noting that, although the questionnaire inquired about the frequency of daytime sleeping, it did not allow for the assessment of nap duration or its timing during the day. In addition, the questionnaire used in the Jeeluna study did not inquire about the age of onset of obesity, and thus did not allow us to examine the association between obesity duration and MHO/MUO status in the study sample. Furthermore, those who consented to blood withdrawal and provided blood samples represented 58.3% of the originally surveyed population. A comparison between those who provided blood samples and those who did not, showed that socio-economic characteristics did not differ significantly between the groups. However, the group that provided blood was older (15.9 ± 1.83 vs. 15.69 ± 1.84 years), heavier (BMI: 22.77 ± 5.92 vs. 22.36 ± 6.06 kg/m^2^), and included more girls compared to boys (50.9% girls vs. 49.1% boys) (*p* < 0.05). Such differences could have resulted in an underestimation of MHO in the study sample, given that BMI has been repetitively shown to be inversely associated with MHO in youth. Despite the above, these differences in age, BMI and gender are less likely to have affected the association between dietary, anthropometric and sleep indicators, as identified in this study. Lastly, the cross-sectional nature of this study does not allow for causality inference. There is a need for longitudinal studies to further confirm the role of adiposity, dietary, psychosocial, socio-demographic and lifestyle- related factors in modulating metabolic profiles in obese youth.

## Conclusion

In a national survey conducted in KSA, this study showed that approximately one out of five adolescents had a favorable metabolic profile, despite being obese. The increasing rate of pediatric obesity underscores the importance of distinguishing MHO and MUO, to optimize the delivery of health services for obesity management in a manner that is both efficient and effective [50]. This study has importantly identified anthropometric factors as predictors of MHO and suggested gender-based differences in the association between diet, sleep and MHO in adolescents. The poor agreement between the two MHO definitions adopted in this study (IDF and CR) and the fact that the use of different definitions yielded different predictors highlight the need for harmonized definitions for the identification of MHO in adolescence. Taken together, the study’s findings provide additional insights into the heterogeneity of obesity, while also having possible impact on intervention strategies aimed at improving metabolic heath in obese adolescents in a region that harbors one of the highest burdens of pediatric obesity worldwide.

## Additional file

Additional file 1: **Table S1.** Psychosocial variables in the total sample of obese adolescents (*n* = 1047) and by MUO or MHO status. (DOCX 17 kb)

## Abbreviations

ATPIII: Adult Treatment Panel III
BMI: Body Mass Index
BP: Blood pressure
CDC: Center for Disease Control and Prevention
CI: Confidence interval
CIMT: Carotid intima media thickness
cm: Centimeters
CR: Cardiovascular risk
CVD: Cardiovascular diseases
d: Day
DBP: Diastolic blood pressure
EMR: Eastern Mediterranean Region
GCC: Gulf Cooperation Council
HDL-C: High density lipoprotein-cholesterol
HOMA: Homeostasis Model Assessment
hrs: Hours
IDF: International Diabetes Federation
IRB: Institutional review board
KAIMRC: King Abdullah International Medical Research Center
kg: Kilograms
kg/m^2^: Kilograms per squared meters
KSA: Kingdom of Saudi-Arabia
LDL-C: Low density lipoprotein-cholesterol
MHO: Metabolically healthy obesity
mm Hg: Millimeters of mercury
mmol/L: Millimoles per liter
mn: Minutes
MOE: Ministry of Education
MUO: Metabolically unhealthy obese
NCD: Non-communicable diseases
NCEP: National Cholesterol Education Program
OR: Odd ratio
SBP: Systolic blood pressure
TC: Total cholesterol
TG: Triglycerides
US: United States
WC: Waist circumference
WHO: World Health Organization
