# Supplementary material for: Prevalence and predictors of metabolically healthy obesity in adolescents: findings from the national “Jeeluna” study in Saudi-Arabia
Source: BMC Pediatr. 2018 Aug 23;18:281. doi: 10.1186/s12887-018-1247-z (PMC6107964; doi:10.1186/s12887-018-1247-z)
Supplement: Supplementary file 1 — Table S1. Psychosocial variables in the total sample of obese adolescents (n = 1047) and by MUO or MHO status. (DOCX 17 kb) [file 12887_2018_1247_MOESM1_ESM.docx]

Additional file 1: Table S1: Psychosocial variables in the total sample of obese adolescents (n=1047) and by MUO or MHO status.

|  | **All** | **IDF** | | | **CR** | | |
| --- | --- | --- | --- | --- | --- | --- | --- |
|  |  | **MUO**  **(n=828)** | **MHO**  **(n=219)** | **p-value** | **MUO**  **(n=798)** | **MHO**  **(n=249)** | **p-value** |
| **Perception of relation with father** |  |  |  |  |  |  |  |
| Not good or average | 160 (15.8) | 126 15.7) | 34 (16.0) | 0.93 | 120 (15.5) | 40 (16.5) | 0.73 |
| Good | 855 (84.2) | 676 (84.3) | 179 (84.0) |  | 652 (84.5) | 203 (83.5) |  |
| **Perception of relation with mother** |  |  |  |  |  |  |  |
| Not good or average | 65 (6.3) | 50 (6.2) | 15 (6.9) | 0.68 | 47 (6.0) | 18 (7.3) | 0.47 |
| Good | 962 (93.7) | 761 (93.8) | 201 (93.1) |  | 734 (94.0) | 228 (92.7) |  |
| **Number of times you have been hit by another student at school (past 12 months** |  |  |  |  |  |  |  |
| ≤1 | 935 (91.5) | 733 (90.8) | 202 (94) | 0.14 | 712 (91.4) | 223 (91.8) | 0.86 |
| >1 | 87 (8.5) | 74 (9.2) | 13 (6.1) |  | 67 (8.6) | 20 (8.2) |  |
| **Number of times you have been hit outside the school (past 12 months)** |  |  |  |  |  |  |  |
| ≤1 | 932 (91.1) | 741 (91.7) | 191 (88.8) | 0.19 | 711 (91.2) | 221 (91) | 0.92 |
| >1 | 91 (8.9) | 67 (8.3) | 24 (11.2) |  | 69 (8.9) | 22 (9.1) |  |
| **Ever felt sad or down for more than 2 weeks during the past 12 months** |  |  |  |  |  |  |  |
| Never, rarely or sometimes | 854 (82.8) | 682 (83.6) | 172 (80.0) | 0.22 | 658 (83.7) | 196 (80.0) | 0.18 |
| Frequently or always | 177 (17.2) | 134 (16.4) | 43 (20.0) |  | 128 (16.3) | 49 (20.0) |  |
| **Ever sought help for feeling down during the past 12 months** |  |  |  |  |  |  |  |
| No | 940 (91.8) | 741 (91.5) | 199 (93) | 0.47 | 715 (91.6) | 225 (92.6) | 0.6 |
| Yes | 84 (8.2) | 69 (8.5) | 15 (7) |  | 66 (8.5) | 18 (7.4) |  |
| **Ever felt worried to a level that affected your daily activities (12 months)** |  |  |  |  |  |  |  |
| Never, rarely or sometimes |  | 760 (93.6) | 200 (93.9) | 0.87 | 732 (93.9) | 228 (93.1) | 0.66 |
| Frequently or always |  | 52 (6.4) | 13 (6.1) |  | 48 (6.2) | 17 (6.9) |  |

Numbers are presented as proportions, n(%).
